# MicroRNA-505 is involved in the regulation of osteogenic differentiation of MC3T3-E1 cells partially by targeting RUNX2

**DOI:** 10.1186/s13018-020-01645-2

**Published:** 2020-04-15

**Authors:** Weihua Li, Zongchao Chen, Chuanqi Cai, Gunjun Li, Xiao Wang, Zhenyu Shi

**Affiliations:** 1 Department of Orthopedics, Huaihe Hospital, Henan University, Kaifeng, 475000 China; 2 Henan Medicial School, Henan University, Jinming Road, Kaifeng, 475004 China

**Keywords:** miR-505, Osteogenic differentiation, MC3T3-E1 cells, RUNX2, miRNA sequence

## Abstract

**Objective:**

Evidence suggests that microRNAs (miRNAs) regulate the expression of genes involved in bone metabolism. This study aimed to investigate the role of miR-505 in the osteogenic differentiation of MC3T3-E1 cells.

**Methods:**

We performed miRNA sequencing to identify differentially expressed miRNAs between MC3T3-E1 cells treated with osteogenic induction medium (OIM) and control cells. Bioinformatics analysis was performed by using the TargetScan and miRDB databases. The expression of miR-505 in MC3T3-E1 cells was detected during osteogenic differentiation. After transfection with miR-505 mimic or miR-505 inhibitor, MC3T3-E1 cells were induced to differentiate into osteoblasts, and the expression of osteogenic differentiation markers (Runt-related transcription factor 2 (RUNX2), alkaline phosphatase (ALP), osteopontin (OPN), osteocalcin (OCN), and osterix (OSX)) was detected.

**Results:**

miR-505 was the most downregulated miRNA among the differentially expressed miRNAs. The RUNX2 gene was identified as a potential target of miR-505 using the target prediction program. miR-505 expression was downregulated during osteogenic differentiation of MC3T3-E1 cells. The expression of osteogenic marker genes was inhibited in MC3T3-E1 cells after transfection with miR-505. However, the expression of osteogenic marker genes was upregulated after transfection with miR-505 inhibitor.

**Conclusion:**

This study is the first to report miR-505 could bind to the RUNX2 gene and thus regulate partly the dysfunction of osteoblasts differentiation, which is expected to be targets for the treatment of osteoporosis.

## Background

Osteoporosis is a chronic systemic bone disease manifesting as lower bone mass, which eventually contributes to increased risk of fractures [1, 2]. Several interacting factors contribute to the risk of osteoporosis and fracture, including hormones, cytokines, transcription factors, and genetic variables. The differentiation of osteoblasts is regulated by many transcription factors, such as Runt-related transcription factor 2 (RUNX2), AMP-dependent transcription factor-4 (ATF4) [3], and osterix (OSX) [4], of which RUNX2 is considered to play a key role in the differentiation of osteoblasts [5]. The abnormal expression of osteoblast genes leads to bone dysplasia or bone formation disorder. The early marker genes of osteogenic differentiation are alkaline phosphatase (ALP), bone sialic acid glycoprotein (BSP), and type I collagen (Col-I), and the late marker genes are osteocalcin (OCN) and osteopontin (OPN).

miRNAs are a class of multifunctional noncoding small RNAs that regulate cell functions and signaling pathways by inhibiting the expression of target mRNAs [6, 7]. miRNAs could regulate gene expression at the post-transcriptional stage by binding to the 3′ untranslated region (UTR) of target mRNAs, which either changes mRNA stability or inhibits protein translation [8]. Several experiments have shown that miRNAs can regulate the function of osteoblasts [9, 10]. Feng et al. [11] reported that miR-152 influences osteoporosis through the regulation of osteoblast differentiation by targeting RICTOR. Yin et al. [12] revealed that miR-135-5p promotes osteoblast differentiation by targeting hypoxia-inducible factor 1 α inhibitor (HIF1AN) in MC3T3-E1 cells. Therefore, exploring the expression of other miRNAs to identify additional targets for osteogenic differentiation could be useful.

To clarify involvement of miRNAs in osteogenic differentiation of MC3T3-E1 cells, we performed microRNA array and found that miR-505 was the significantly differentially expressed miRNA. In experiment study, we found that miR-505 was downregulated in the process of osteogenic differentiation of MC3T3-E1 cells. Moreover, we also determined that miR-505 could directly bind to RUNX2 and thus inhibit osteogenic differentiation.

## Materials and methods

### Reagents

The following reagents were used in this study: α-MEM medium (HyClone, USA); fetal bovine serum (FBS) and Opti-MEM medium (Gibco, USA); 0.25% trypsin, dexamethasone, ascorbic acid, and β-glycerol phosphate (Sigma, USA); Lipofectamine RNAiMAX reagent, TRIzol reagent, and protein marker (Thermo Fisher, USA); PrimeScript RT reagent Kit with gDNA Eraser kit and SYBR Green PCR Master Mix (Bao Bioengineering Co., Ltd. ); miRNA quantitation and U6 calibration qRT-PCR kit, miR-505 mimics, inhibitor, and negative control (NC) (Suzhou Jima Gene Co., Ltd.); Alizarin red staining reagent (Saiye Biotechnology Co., Ltd. ); RUNX2, OSX, OPN, ALP, GAPDH, miR-505, and U6 primers (AUGCT, Beijing, China); BCA protein assay kit (Bioengineering Co., Ltd.), RIPA lysis buffer, and SDS-PAGE Gel configuration Kit (Biyuntian Biotechnology Co., Ltd.); PVDF membrane and chemiluminescence ECL solution (Millipore, USA); anti-OPN, anti-OCN, and anti-GAPDH antibodies (Santa Cruz Biotechnology, USA); anti-RUNX2 antibody (CST, USA); anti-ALP antibody (Abcam, USA); and other reagents generated by our group.

### Cell lines and osteogenic induction

Mouse progenitor osteoblasts MC3T3-E1 were purchased from the Cell Bank of the Chinese Academy of Sciences. The cell culture medium was 10% FBS in MEM containing 0.35 g/L glutamine. The cells were cultured in a constant-temperature cell culture incubator at 5% CO_2_ and 37 °C, and the cells were subcultured once a day. A single cell suspension was prepared from MC3T3-E1 cells growing under steady-state conditions and inoculated in a 6-well culture plate. The density of MC3T3-E1 cells was more than 80% after plating in a 6-well culture plate, and the osteogenic induction medium (OIM, α-MEM containing 10% FBS, 10^−7^ mol/L dexamethasone (Sigma-Aldrich, D1756), 50 μg/mL ascorbic acid (Sigma-Aldrich, 795437), and 10 mmol/L β-glycerophosphate sodium (Sigma-Aldrich, G9422)) was replaced every 2 days.

### miRNA sequencing

The RiboArray miDETECT mouse array (RiboBio Co., Guangzhou, China) was used to detect the microRNA expression levels in 3 independent replicates for MC3T3-E1 without osteoblast induction and 3 independent replicates for MC3T3-E1 which underwent for 10 days in OIM. Briefly, MC3T3-E1 cells (2 × 10^5^ cells per well) were initially seeded in a six-well plate, at 70% confluence; the cells were treated with growth medium (control) or OIM for 10 days, respectively.

Then, total RNA in the control and OIM groups was extracted by TRIzol (Invitrogen) according to the manufacturer’s instructions. NanoDrop ND-1000 (Thermo) was used to measure the concentration of purified RNA and detect the quality of RNA. RNA labeling, microarray chip hybridization, and scanning were performed according to the method and instructions provided by the Affymetrix Multispecies miRNA-2 Array.

### Bioinformatics analysis of miR-505

First, we predicted miR-505 targets using the TargetScan (http://www.targetscan.org/vert_71/) and miRDB (http://mirdb.org) databases. Then, a Venn diagram of the results from these two databases was generated using Venny 2.1 (http://bioinfogp.cnb.csic.es/tools/venny/). Overlapping genes were then submitted to DAVID 6.8 (https://david.ncifcrf.gov/tools.jsp) to identify Gene Ontology (GO) categories (biological process (BP), molecular function (MF), and cellular component (CC)) and perform KEGG pathway analysis.

### Real-time PCR analysis

Total RNA extracted from the cells was quantified by a microspectrophotometer at 0, 3, 7, and 10 days after osteogenic induction. cDNA was synthesized by reverse transcription reaction according to the instruction of the Hairpin-it miRNA quantitation and U6 calibration qRT-PCR kit, and then, fluorescence quantitative PCR amplification was carried out according to the instructions. The amplification program was as follows: 95 °C, 3 min predenaturation; 95 °C, 12 s; and 60 °C, 40 s for 40 cycles. After the reaction, the cycle threshold number (Ct value) of each specimen and the internal reference gene U6 were obtained. The relative content of miR-505 was calculated relative to housekeeping U6 with the 2^−ΔΔCt^ equation [13]. The relative expression of RUNX2, OSX, OPN, and ALP was calculated relative to housekeeping GAPDH with the 2^−ΔΔCt^ equation [14]. The experiment was repeated three times. The sequence of primers used in this experiment is shown in Table 1.

**Table 1:** Primers used for reverse transcription and PCR in this study

Gene	Primer sequence (5′–3′)
RUNX2	F: CTCTTCCCAAAGCCAGAGTC
	R:CAGCGTCAACACCATCATTC
OSX	F: GAGGAAGAAGCCCATTCACA
	R:GCAGGCAGGTGAACTTCTTC
ALP	F: AACCCAGACACAAGCATTCC
	R:GCCTTTGAGGTTTTGGGTCA
OPN	F:GACGATGATGACGATGG
	R:CCTCAGTCCATAAGCCAAGC
GADPH	F:AAGGTCATCCCAGAGCTGAA
	R:AGGAGACAACCTGGTCCTCA
U6	F:ATTGGAACGATACAGAGAAGATT
	R:AGGAACGCTTCACGAATTTG
miR-505	F:GCGGGAGCCAGGAAGTAT
	R:CAGTGCGTGTCGTGGAGT

### Alizarin Red S (ARS) staining

MC3T3-E1 cells were induced to undergo osteogenesis for 21 days, after which the culture medium was discarded, and the cells were washed with PBS 2 times, fixed with 4% paraformaldehyde for 30 min, washed with PBS 2 times, stained with alizarin red 10 min, washed completely with PBS, and observed by inverted microscopy.

### Cell transfection and osteogenic induction culture

MC3T3-E1 cells were digested by trypsin and resuspended in α-MEM containing 10% FBS. The cells were inoculated in 6-well culture plates at a density of 2.5 × 10^5^/mL. MC3T3-E1 cells were randomly divided into 4 groups: miR-505 mimics, miR-505 mimics-NC, miR-505 inhibitor, and miR-505 inhibitor-NC. Lipofectamine RNAiMAX liposomes were used for transfection. Then, miRNA duplexes at a concentration of 30 nmol/L were mixed with 150 μL Opti-MEM medium. Concurrently, Lipofectamine RNAiMAX reagent was mixed with 150 μL Opti-MEM medium at 7.5 μL per well. After incubation for 5 min, the miRNA and Lipofectamine RNAiMAX solutions were mixed and incubated at room temperature for 20 min. The medium in the 6-well plate was discarded, and 700 μL Opti-MEN culture medium was added to each well. Then, the incubated miRNA/Lipofectamine RNAiMAX mixture was added to each well and gently mixed. After the cells were incubated in a constant-temperature cell culture incubator at 37 °C for 6 h, α-MEM with 10% FBS was added, and the cells continued to be cultured. Then, the osteogenic induction medium was added for 3 days.

### Cell transfection and identification

To determine the success of miR-505 transfection, the expression of miR-505 was detected by real-time quantitative RT-PCR. After transfection with 15, 30, or 60 nmol/L concentration of miR-505 mimics, miR-505 mimics-NC, miR-505 inhibitor, or miR-505 inhibitor-NC for 24 h, total RNA was extracted to detect the mRNA expression and miR-505 expression levels. Optimal concentration was identified as used for further experiments. All of the transfections were performed by using Lipofectamine 3000 (L3000001; Thermo Fisher Scientific, USA) based on the provided directions [15].

The cells were transfected with miR-505 mimics, miR-505 mimics-NC, miR-505 inhibitor, or miR-505 inhibitor-NC for 24 h and cultured for 3 additional days after replacement of the medium with osteogenic induction medium. The mRNA expression of RUNX2, OSX, ALP, and OPN in cells was detected by collecting and extracting total RNA.

### Western blot to detect the regulatory effect of miR-505 on osteogenic differentiation

MC3T3-E1 cells were transformed with miR-505 mimics, miR-505 mimics-NC, miR-505 inhibitor, or miR-505 inhibitor-NC and induced to undergo osteogenesis for 3 days. Total protein was collected and lysed with RIPA buffer. The protein concentration was determined by BCA protein quantitative test. The total protein was transferred to a PVDF membrane by SDS-PAGE. After blocking with skimmed milk powder, 1:1000-diluted anti-RUNX2, anti-OPN, anti-OCN, anti-ALP, and anti-GAPDH antibodies were added overnight at 4 °C. After rinsing, the secondary antibody was incubated at 1:2000, at room temperature for 2 h.

### Luciferase reporter gene assay

MC3T3-E1 cells were firstly inoculated into 12-well plates at a density of 1 × 10^5^ cells per well. 3′ UTR of the RUNX2 gene containing putative miR-505 targeting site was amplified by chemical synthesis and was inserted into the psiCHECK2 vector (Promega, Madison, WI, USA). When the confluence was up to 70%, MC3T3-E1 were transfected with related mixtures including 50 ng RUNX2 wild-type or RUNX2 mutant-type 3′ UTR reporter plasmids, miR-505 mimics or miR-505 mimics NC in a final concentration of 20 nM, and Lipofectamine 2000 for 48 h. Luciferase activity was detected using the dual-luciferase reporter gene kit (Beyotime, Shanghai, China).

### Statistical analysis

The experimental data are expressed as the mean ± standard deviation. Differences between two groups were analyzed by two-sided Student’s *t* test. The results were considered to be statistically significant when *P* < 0.05. All statistical analyses were performed using SPSS 19.0 (IBM Corp., Armonk, NY, USA).

## Results

### Preliminary screening by miRNA array chip

**Fig. 1:**
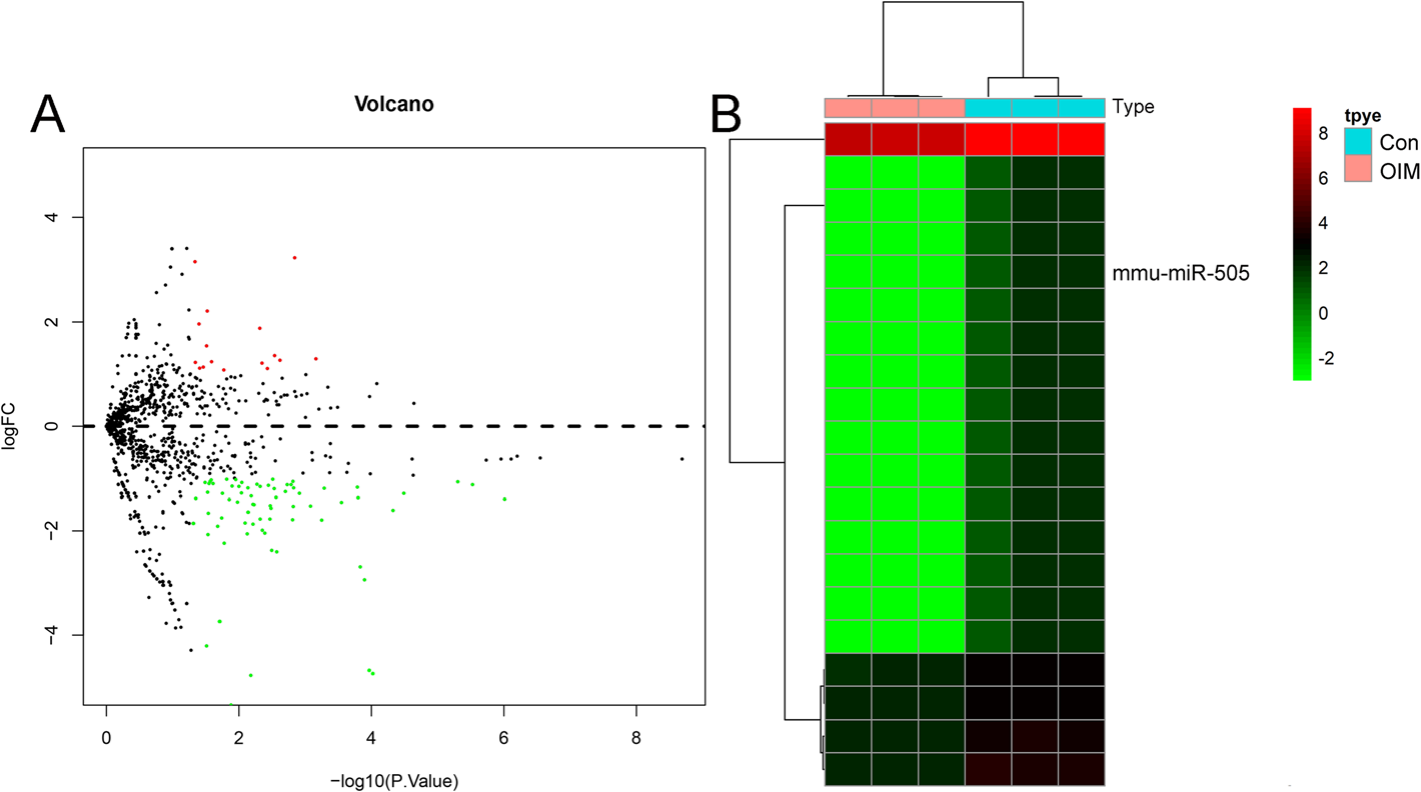
**a** Volcano plot of the differentially expressed miRNAs between osteogenic and control groups. **b** Heatmap of the differentially expressed miRNAs in control (Con) and osteogenic induction medium (OIM) groups

We used volcano plot to show the inter-relationships between differentially expressed mRNAs in MC3T3-E1 cells in the control (Con) and OIM groups (Fig. 1a). One hundred and forty-eight differentially expressed miRNAs were identified through initial microarray chip analysis. Among them, 18 were significantly upregulated, and 130 were significantly downregulated (fold change ≥ 2.0, *P* value ≤ 0.05). Among the differentially expressed miRNAs, miR-550 was the most downregulated miRNA. A heatmap of differentially expressed miRNAs between Con and OIM groups is shown in Fig. 1b.

### Bioinformatics analysis

**Fig. 2:**
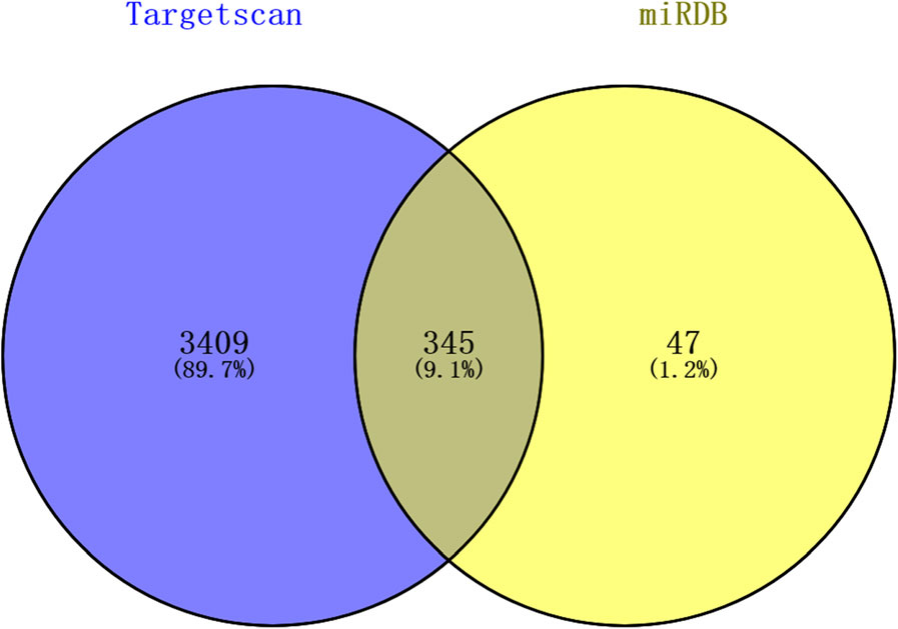
Venn diagram of the overlapping genes of the TargetScan and miRDB results

**Fig. 3:**
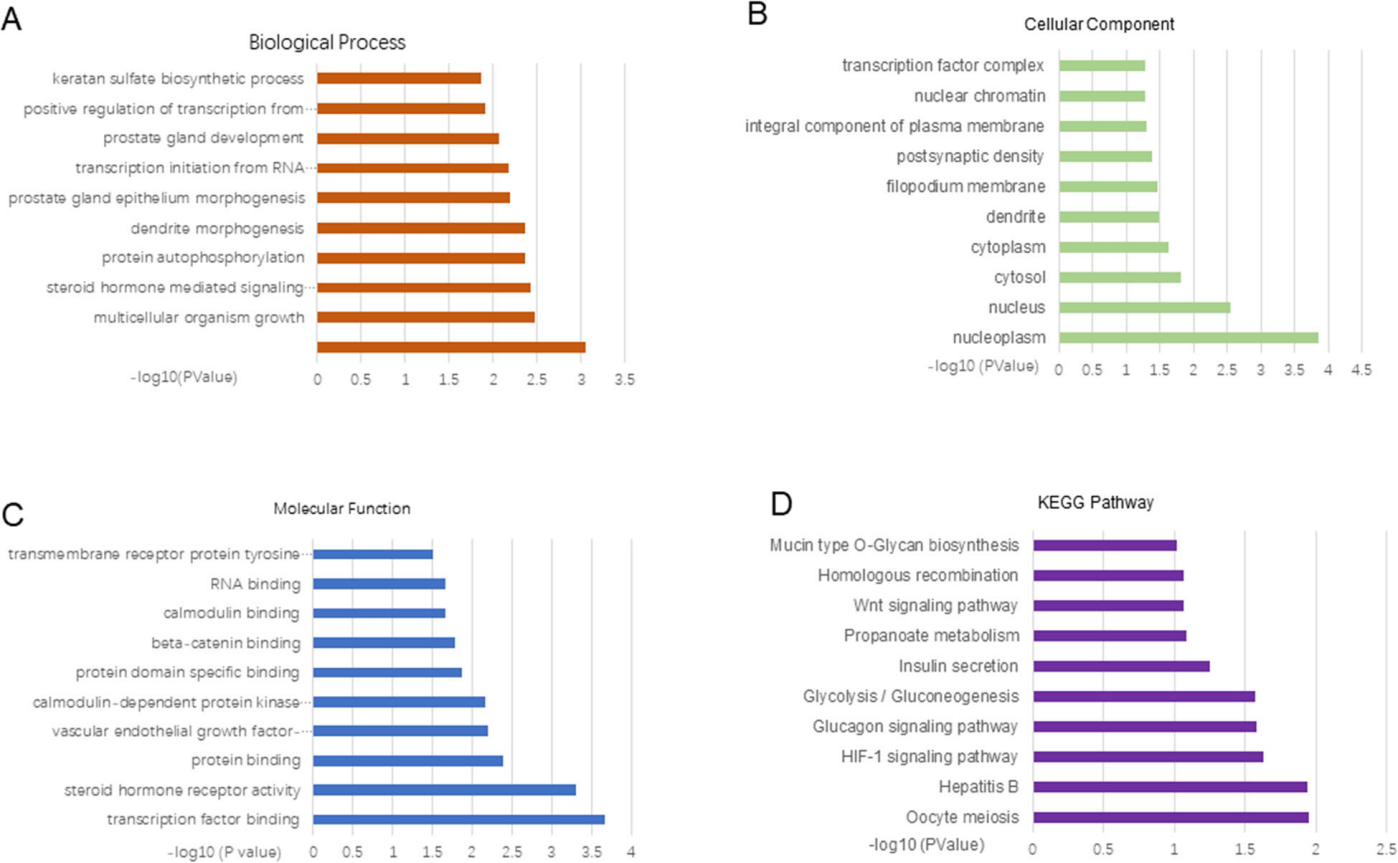
**a** Biological process terms of the predicted genes. **b** Cellular component terms of the predicted genes. **c** Molecular function terms of the target genes. **d** KEGG pathway of the predicted genes

First, a total of 3754 and 392 target genes were predicted in the TargetScan and miRDB databases, respectively. There were a total of 345 overlapping genes between these two databases (Fig. 2). Figure 3 shows the top ten GO categories and KEGG pathways. We found that the predicted genes were most enriched in multicellular organism growth (Fig. 3a, biological process), and nucleoplasm (Fig. 3b, cellular component) and transcription factor binding (Fig. 3c, molecular function). The most enriched KEGG pathway was oocyte meiosis (Fig. 3d).

### Changes in miR-505 expression during osteogenic differentiation

**Fig. 4:**
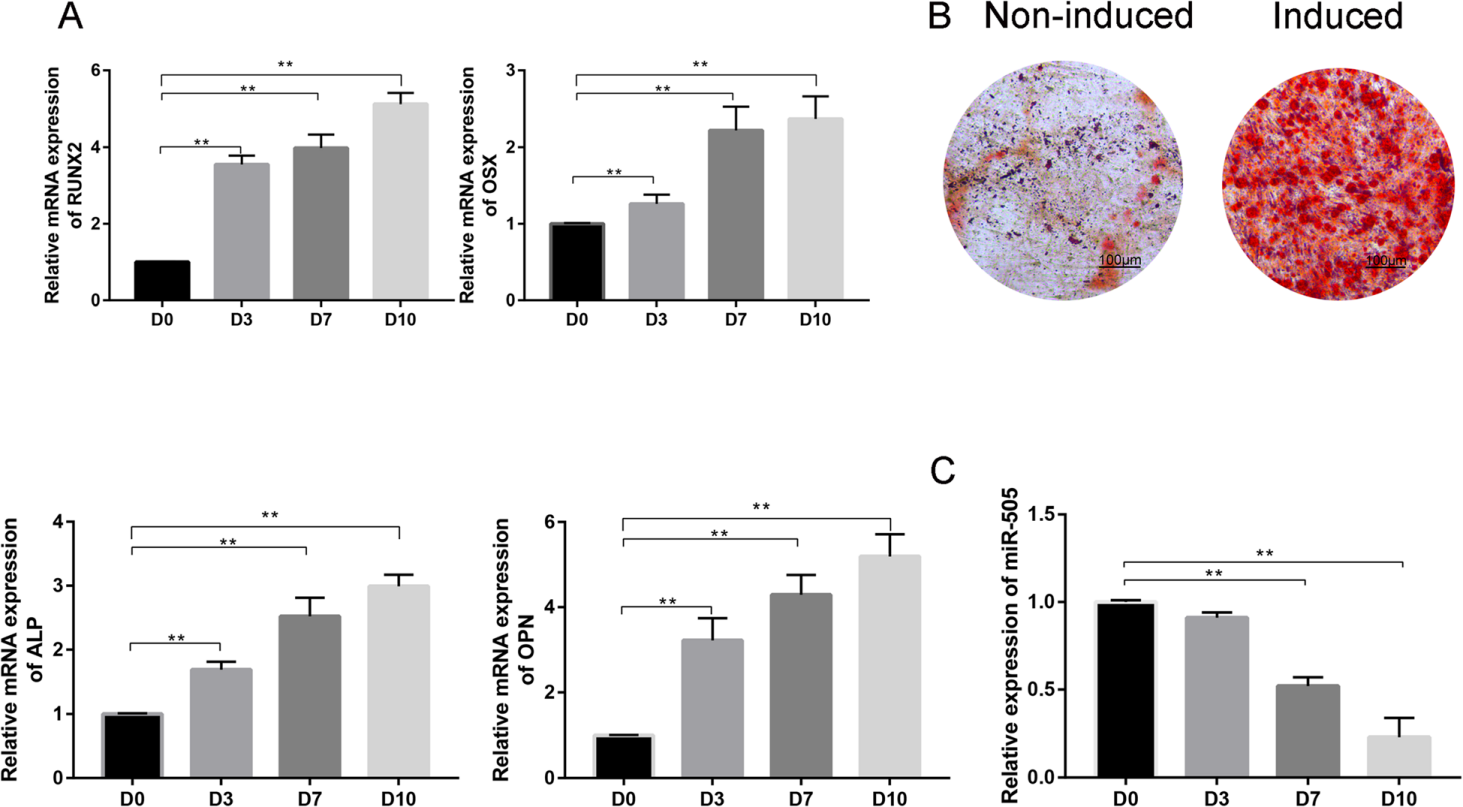
**a** Relative mRNA expression of RUNX2, OSX, ALP, and OPN during the osteogenic differentiation of MC3T3-E1 cells (from day 0 to day 10). **b** Alizarin Red S (ARS) staining between the non-induced and induced groups. **c** Relative expression of miR-505 during the osteogenic differentiation of MC3T3-E1 cells (from day 0 to day 10)

MC3T3-E1 cells were induced by osteogenic differentiation medium for 10 days. The results showed that the mRNA expression of RUNX2, OSX, ALP, and OPN in the cells increased with increasing induction time (Fig. 4a). In addition, the cells cultured in osteogenic induction medium for 10 days also showed a large number of red calcified nodules deposited with mineral salt (Fig. 4b), indicating that the process of osteogenic differentiation of MC3T3-E1 cells was successful. RT-PCR was used to detect the expression of miR-505 during osteogenic differentiation. The results showed that the expression of miR-505 decreased gradually with increasing osteogenic induction time, and there was a significant difference between the 7th and 10th day compared to the 0th day (*P* < 0.001, Fig. 4c).

### RT-PCR was used to detect the expression of miR-505 in each group

**Fig. 5:**
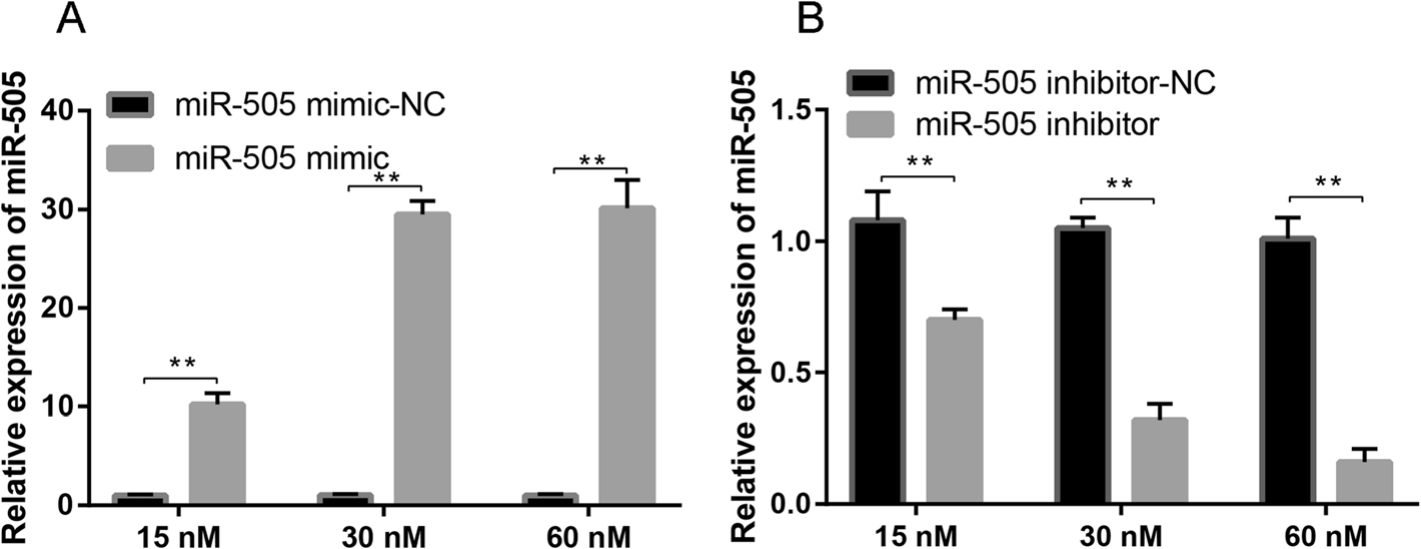
Transfection efficiencies in the 15-nM, 30-nM, and 60-nM groups after transfection with miR-505 mimic (**a**) or inhibitor (**b**)

The expression of miR-505 transfected at concentrations of 15, 30, or 60 nmol/L in MC3T3-E1 cells was detected by RT-PCR. The results showed that the expression of miR-505 in MC3T3-E1 cells of the miR-505 mimic group was significantly upregulated in a concentration-dependent manner compared with that in the corresponding NC group. The higher the concentration of miR-505 mimics, the higher the expression of miR-505, and the difference was statistically significant (Fig. 5a, *P* < 0.001). Compared with that in miR-505 inhibitor-NC-treated cells, the expression of miR-505 in miR-505 inhibitor-treated cells was significantly downregulated in a concentration-dependent manner (Fig. 5b, *P* < 0.001), and the expression of miR-505 was also downregulated in a concentration-dependent manner (Fig. 5b, *P* < 0.05).

### Effect of miR-505 on osteogenic differentiation of MC3T3-E1 cells

**Fig. 6:**
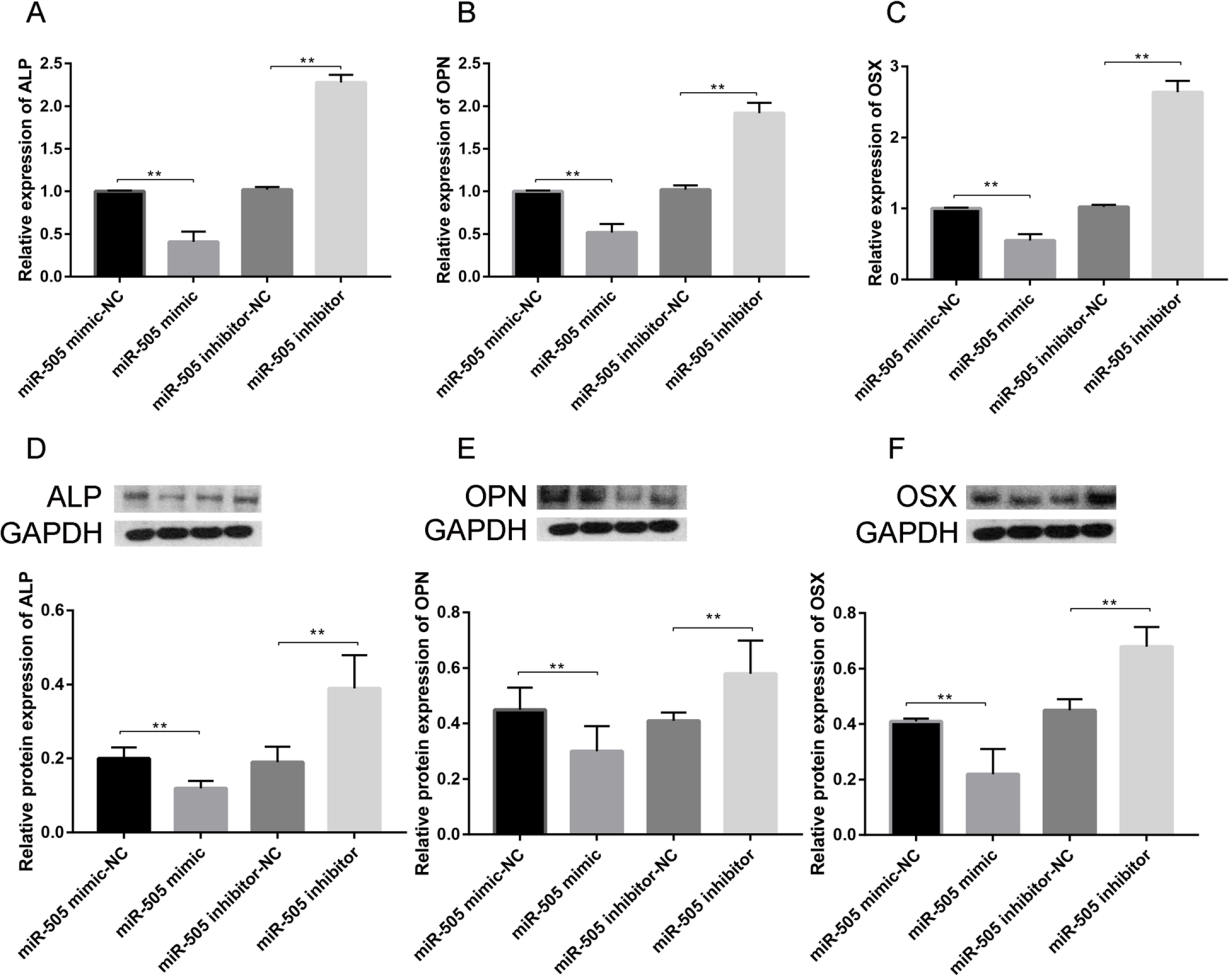
Effect of miR-505 on the expression of osteogenic differentiation-related genes (**a** ALP, **b** OPN, and **c** OSX) and protein expression (**d** ALP, **e** OPN, and **f** OSX)

To verify the effect of miR-505 on the osteogenic differentiation of MC3T3-E1 cells, miR-505 mimics, miR-505 mimics-NC, miR-505 inhibitor, and miR-505 inhibitor-NC were transfected into cells at a final concentration of 30 nmol/L, and then, the cells were treated with osteogenic induction medium for 3 days. The expression of osteogenic-related genes was detected by RT-PCR and Western blot. The results showed that compared with that in the miR-505 mimics-NC group, the mRNA (Fig. 6a–c) and protein expression (Fig. 6d–f) levels of the ALP, OPN, and OSX genes in the miR-505 mimics group were significantly decreased.

### miR-505 modulates osteogenesis by targeting the RUNX2 gene

**Fig. 7:**
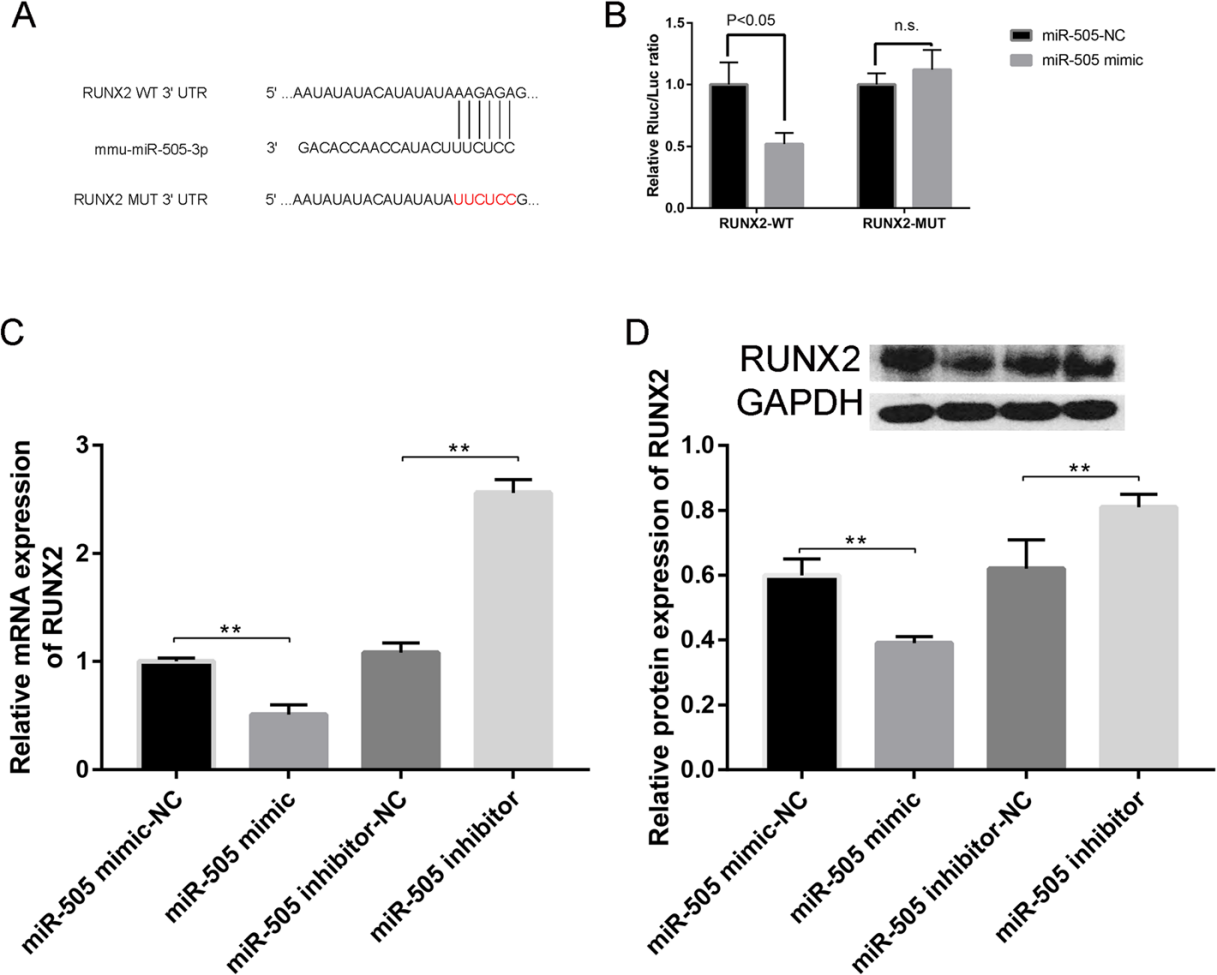
**a** Targeting site of miR-505 in RUNX2. **b** Relative Rluc/Fluc ratio of RUNX2 expression. **c** Relative mRNA expression of RUNX2 in cells treated with miR-505 mimic, miR-505 mimic-NC, miR-505 inhibitor-NC, or miR-505 inhibitor. **d** Relative protein expression in cells transfected with miR-505 mimic-NC, miR-505 mimic, miR-505 inhibitor-NC, and miR-505 inhibitor

The predictive complementary sequences are shown in Fig. 7a. The luciferase reporter gene assay in Fig. 7b further showed that the combination of RUNX2-WT and miR-505 mimic largely decreased fluorescence intensity compared with the combination of RUNX2-WT and miR-505 mimic NC.

To further determine whether miR-505 can regulate osteogenic differentiation by targeting the RUNX2 gene, we detected the expression of the Runx2 gene by RT-PCR after transfection with miR-505 mimics or miR-505 inhibitor. The results showed that compared with that in the miR-505 mimic-NC group, the mRNA expression level of the RUNX2 gene in cells overexpressing miR-505 was significantly downregulated, while the expression level of the RUNX2 gene in the miR-505 inhibitor group was significantly upregulated (Fig. 7c). In addition, the protein expression of RUNX2 was consistent with the mRNA results, suggesting that miR-505 regulated osteogenic differentiation by targeting the RUNX2 gene (Fig. 7d).

## Discussion

In this study, we found that miR-505 was downregulated during osteogenic differentiation of MC3T3-E1 cells. Moreover, miR-505 negatively regulated osteogenic differentiation of MC3T3-E1 cells by targeting RUNX2.

Osteoporosis is a relatively prevalent disease that mainly leads to loss of bone mass and microstructural deterioration of bone tissues and eventually increases the susceptibility of patients to bone fractures [16, 17]. It has been found that abnormal expression of many genes can cause osteoporosis, among which RUNX2 is the key transcription factor regulating osteoblast differentiation and bone formation during bone development [18, 19]. RUNX2 is involved in the regulation of bone metabolism through a variety of pathways, but its specific mechanism is not clear [20].

In this study, we found that miR-505 was downregulated during osteogenic differentiation of MC3T3-E1 cells. Moreover, we found that miR-505 directly binds to the RUNX2 3′ UTR, thus inhibiting the osteogenic differentiation of MC3T3-E1 cells. Kapora et al. [21] demonstrated that miR-505 functions as a tumor suppressor by targeting cyclin-dependent kinase 5 in cervical cancer. Ren et al. [22] revealed that miR-505 suppressed the growth of hepatocellular carcinoma cells by targeting IGF-1R.

A large number of studies have confirmed that miRNAs are involved in the regulation of osteoblast differentiation. miR-29a modulates the expression of RUNX2 in osteoblasts by regulating the Wnt signaling pathway, ERK1/2 pathway, and Akt phosphorylation [23]. Sun et al. [24] found that miR-503 promotes bone formation in distraction osteogenesis by suppressing smurf1 expression. Lin et al. [25] revealed that miR-130a controls bone marrow mesenchymal stem cell differentiation towards an osteoblastic and adipogenic fate. Until now, there has been no study on the role of miR-505 in the osteogenic differentiation of MC3T3-E1 cells.

Software prediction analysis showed that miR-505 could target the RUNX2 gene sequence. After overexpression of miR-505 mimics in MC3T3-E1 cells, the expression of RUNX2 was downregulated, suggesting that RUNX2 could be a target gene of miR-505. The RUNX2 gene is a landmark gene of osteogenic differentiation and bone formation [26]. RUNX2 can activate the transcription and expression of OPN, ALP, and OCN [27]. After overexpression of miR-505 in MC3T3-E1 cells, the expression of the osteogenic marker genes ALP, OCN, and OPN was inhibited, while the osteogenic marker genes were regulated to varying degrees after miR-505 inhibitor.

## Conclusion

In summary, the results obtained in the present study indicated that miR-505 was downregulated during osteogenic differentiation in vitro and that its overexpression could inhibit osteogenic differentiation and suppress the growth of osteoblast cells by targeting RUNX2. Therefore, miR-505 may be a promising therapeutic target for the promotion of new bone regeneration.

## Data Availability

We declare that the materials described in the manuscript will be freely available to all scientists for non-commercial purposes.

## Abbreviations

ALP: Alkaline phosphatase
ATF4: AMP-dependent transcription factor-4
BP: Biological process
BSP: Bone sialic acid glycoprotein
CC: Cellular component
Col-I: Type I collagen
FBS: Fetal bovine serum
GO: Gene Ontology
HIF1AN: Hypoxia-inducible factor 1 α inhibitor
MF: Molecular function
miRNAs: MicroRNAs
NC: Negative control
OCN: Osteocalcin
OIM: Osteogenic induction medium
OPN: Osteopontin
OSX: Osterix
RUNX2: Runt-related transcription factor 2
